# Serum biochemical parameters and cytokine profiles associated with natural African trypanosome infections in cattle

**DOI:** 10.1186/s13071-017-2255-9

**Published:** 2017-06-27

**Authors:** Soale Majeed Bakari, Jennifer Afua Ofori, Kwadwo Asamoah Kusi, George Kwame Aning, Gordon Akanzuwine Awandare, Mark Carrington, Theresa Manful Gwira

**Affiliations:** 1 West African Centre for Cell Biology of Infectious Pathogens, College of Basic and Applied Sciences, University of Ghana, Legon, Ghana; 2 Department of Biochemistry, Cell and Molecular Biology, College of Basic and Applied Sciences, University of Ghana, Legon, Ghana; 3 Department of Immunology, Noguchi Memorial Institute for Medical Research, College of Health Sciences, University of Ghana, Legon, Ghana; 4 School of Veterinary Medicine, College of Basic and Applied Sciences, University of Ghana, Legon, Ghana; 5 Department of Biochemistry, University of Cambridge, Cambridge, UK

**Keywords:** Animal African trypanosomiasis, Natural infections, Cytokine profiles, Serum biochemical parameters

## Abstract

**Background:**

Animal African trypanosomiasis (AAT) greatly affects livestock production in sub-Saharan Africa. In Ghana prevalence of AAT is estimated to range between 5 and 50%. Studies have reported serum biochemical aberrations and variability in cytokine profiles in animals during infection. However, information regarding the biochemical parameters and cytokine profiles associated with natural infections are limited. This study was therefore aimed at investigating changes in the levels of serum biochemical parameters and inflammatory cytokines during a natural infection.

**Methods:**

Nested internal transcribed spacer (ITS)-based PCR and sequencing were used to characterise trypanosome infection in cattle at two areas in Ghana (Adidome and Accra) of different endemicities. The cattle were sampled at four to five-week intervals over a period of six months. Levels of serum biochemical parameters, including creatinine, cholesterol, alkaline phosphatase (ALP), alanine aminotransferase (ALT), total bilirubin and total protein and cytokines (interleukin 10, interleukin 4, interleukin 12, interferon gamma and tumor necrosis factor alpha) were measured in serum samples and then compared between infected cattle and uninfected controls.

**Results:**

The predominant trypanosome species detected in Accra (non-endemic) and Adidome (endemic) were *Trypanosoma theileri* and *Trypanosoma vivax*, respectively. Serum biochemical parameters were similar between infected and uninfected cattle in Accra. Infected cattle at Adidome however, had significantly higher levels of ALP, creatinine, total protein and total bilirubin (*P* < 0.05) and significantly lower levels of cholesterol (*P* < 0.05) at specific time points. At basal levels and during infection, significantly higher pro-inflammatory to anti-inflammatory (Th1/Th2) cytokine ratios were observed in cattle at Adidome compared to Accra (*P* < 0.05), indicating a shift towards Th1 immune response in Adidome. Levels of IL-10 were, however, significantly elevated in infected cattle in Accra (*P* < 0.05), suggesting high anti-inflammatory cytokine response in Accra.

**Conclusion:**

These results suggests that cattle in an endemic area repeatedly infected with trypanosomes of different species or different antigenic types demonstrate high pro-inflammatory (Th1) immune response and biochemical alterations whereas cattle in a non-endemic area with predominantly chronic *T. theileri* infections demonstrate high anti-inflammatory response and no biochemical alterations.

**Electronic supplementary material:**

The online version of this article (doi:10.1186/s13071-017-2255-9) contains supplementary material, which is available to authorized users.

## Background

Animal trypanosomiasis remains a major constraint to livestock production in sub-Saharan Africa despite various strategies employed to control, treat and eradicate the disease. The disease is caused by several species of trypanosomes, and the species and strain of parasite can have great influence on the severity and course of infection, as well as on the epidemiology of host-parasite-vector relationships [1]. The major pathogenic trypanosome species responsible for causing disease in animals are *Trypanosoma congolense*, *T. vivax*, *T. brucei brucei* and *T. simiae* [2]. Infection is usually chronic, characterised by intermittent fever, anaemia, lymphadenopathy and weight loss [3, 4]. During infection, trypanosomes persist in the host bloodstream, evading immune responses and invading vital body organs such as liver, spleen and kidneys [5, 6]. Various pathological disorders result following trypanosome infection. Abnormalities in mammalian host physiology such as changes in blood pH, hormones, nutrients and levels of metabolites such as cholesterol and creatinine occur through the course of infection in cattle, dogs, pigs, sheep and goats [6–11]. Studies have also revealed significant increases in serum enzymes such as aspartate aminotransferase (AST), alanine aminotransferase (ALT) and alkaline phosphatase (ALP) in experimental trypanosome infections, suggestive of tissue/organ damage [7, 12, 13]. There are a few conflicting reports on serum biochemical changes in mammals due to trypanosome infection; however, haematological, pathological and serum biochemical aberrations are associated with infection [6]. Serum biochemical and haematological parameters, therefore, constitute important indices for disease diagnosis and prognosis [14].

Immune response to trypanosome infections is mediated by various cytokines. A shift from the production of pro-inflammatory cytokines at the beginning of infection to anti-inflammatory cytokines in late or chronic infection is correlated with the ability of the host to reduce parasitaemia and pathology, respectively [15]. Namangala et al. [16] observed in mouse models that a balance between pro- and anti-inflammatory cytokines is central to the outcome of the disease. Previous studies have demonstrated the production of interferon-gamma (IFN-γ), interleukin 12 (IL-12), nitric oxide (NO) and tumour necrosis factor alpha (TNF-α) in early stages of infection and later interleukin 13 (IL-13), interleukin 4 (IL-4) and interleukin 10 (IL-10) [15, 17, 18]. IL-10 and IL-6 have been shown to be upregulated in late-stage human African trypanosomiasis [19]. Due to upregulation of different types of cytokines at different stages of infections, cytokines have been proposed as potential diagnostic or stage biomarkers [20]. However, information on the cytokine profiles in animals during natural trypanosome infection is limited. Most of the studies on biochemical parameters and cytokines have been carried out on experimentally infected animals with usually a single species of trypanosome. Also, the studies have been mainly cross-sectional and hence do not show how serum biochemical parameters change over a period of infection and whether there are changes in the levels and types of cytokines as infection persists under natural conditions. Hence, the extent of alterations caused by natural trypanosome infection in animals and the cytokines involved in the immune responses at different stages of natural infection are not fully understood.

This study was therefore carried out to longitudinally characterise animal African trypanosomes and determine the associated levels of serum biochemical parameters and cytokine profiles in naturally infected cattle in Ghana over a six-month period.

## Methods

### Study sites and animal population

The study was conducted at two separate sites of different endemicity: the University of Ghana Livestock and Poultry Research Farm at Accra in the Greater Accra region, and a cattle ranch at Adidome in the Volta region. Accra is in the coastal savanna vegetation zone, has a low tsetse fly density and low prevalence of animal trypanosomiasis. Adidome is located 130 km east of Accra, in the transitional forest vegetation zone, with a high tsetse fly density and a high prevalence of animal trypanosomiasis. The study population included cattle aged between 6 to 18 months, irrespective of the sex and breed. A total of 40 cattle, 20 from each of the study locations were randomly selected and ear-tagged. Additional file 1: Table S1 contains information on the age, sex and breeds of cattle selected at the two study areas. The selected cattle at Accra were of Sanga, Sanga cross and West African Shorthorn (WASH) breeds, whereas at Adidome, the cattle used were of the Sanga cross breed.

### Sample collection

Peripheral blood samples were collected from cattle at the two study sites at four to five-week intervals over six different time points. Two blood samples (5 ml each) were collected by jugular venipuncture from each animal into S-monovette blood collection tubes (Sarstedt AG & Co, Nümbrecht, Germany), one coated with EDTA and the other without the additive. Samples in EDTA tubes were placed on ice during transportation.

### Isolation and purification of DNA from whole blood samples

DNA was extracted from uncoagulated whole blood samples (blood in EDTA tubes) using the QIAamp DNA Blood Maxi Kit (Spin Protocol) (QIAGEN, Hilden, Germany), following the manufacturer’s protocol. The extracted DNA was further purified using the QIAquick PCR Purification Kit (QIAGEN), following the manufacturer’s instructions.

### Serum separation

Blood samples in S-monovette tubes without additive were allowed to clot and serum separated by centrifugation at 3000× *rpm* for 10 min. The separated sera were then aliquoted into four 1.5 ml Eppendorf tubes for each sample and then stored at −80 °C until used. The processing of the serum samples was carried out 3–4 h after sample collection.

### Detection of trypanosomes by nested ITS-based PCR

Trypanosome DNA was amplified by a nested ITS-based PCR technique as described by Cox et al. [21], with slight modifications. For the first round of PCR, 1 μl of the purified whole blood DNA was added to a PCR reaction mixture of 29 μl. The reaction mixture contained 1× Mango *Taq* buffer with 1.5 mM MgCl_2_, 0.3 μM outer primers TM1 (5′-GAT TAC GTC CCT GCC ATT TG-3′) and TM2 (5′-TTG TTC GCT ATC GGT CTT CC-3′), 0.3 mM dNTP’s and 2.5 U Mango *Taq* polymerase. The thermal cycling was carried out under the following reaction conditions; 1 cycle of 95 °C for 5 min followed by 30 cycles of 95 °C for 30 s, 55 °C for 30 s, 72 °C for 1 min, and then 1 cycle of 72 °C for 5 min followed by cooling at 4 °C. For the second round of PCR reaction (nested step), 1 μl of the PCR product from the first round reaction was placed in a fresh tube, and 29 μl of the reaction master mix added. The reaction mixture had the same components as the reaction mix for the first PCR reaction except for the outer primers which were replaced with inner primers TM3 (5′-GGA AGC AAA AGT CGT AAC AAG G-3′) and TM4 (5′-TGT TTT CTT TTC CTC CGC TG-3′). The cycling conditions used were the same as that for the first PCR reaction. After the amplification, 10 μl of the PCR products were run on a 1.2% agarose gel at 100 V for 1 h. The ethidium bromide stained gel was then visualised under ultraviolet (UV) light using a UV transilluminator. PCR products from positive samples were gel purified using the QIAquick gel extraction kit (QIAGEN) following the manufacturer’s instructions, and then sequenced.

### Measurement of serum biochemical parameters

The serum levels of glucose, total serum protein, bilirubin, creatinine, total cholesterol, alkaline phosphatase and alanine aminotransferase were determined using commercial reagent kits (ELITech Group, Puteaux, France) and Flexor E automated clinical chemistry analyser (Vital Scientific NV, Dieren, The Netherlands).

### Quantification of serum cytokines in cattle

Serum concentrations of IL-10, IL-4, IFN-γ, TNF-α and IL-12 were measured using commercially available sandwiched bovine cytokine ELISA kits (Cusabio Biotech Co., Ltd., Wuhan, China), following the manufacturers’ protocols. Samples from time point 1 at Accra and time point 4 at Adidome were selected for the cytokine quantification due to representative numbers of infected and uninfected cattle at these time points. Cytokines were also quantified in cattle that had the same state of infection (infected throughout the study period or uninfected throughout the study period) over the study period using samples from each of the time points. All samples were run in duplicate and concentrations of all the cytokines extrapolated from calibration curves constructed for the bovine cytokine standards using the measured optical densities (ODs) in an Auditable Data Analysis and Management System for ELISA (ADAMSEL).

### Data analysis

Data obtained were analysed using STATA 13.0 statistical software (StataCorp LP, Texas, and USA) and GraphPad Prism (version 6, San Diego, CA, USA). Since the data for the various groups compared in the study were largely non-parametric, Mann-Whitney U-test was used for comparison of medians between these groups at each time point. At each study site, the potential effect of animal age on the measured biochemical parameters and cytokines were assessed by comparing the median ages of infected and uninfected animals at each time point by Mann-Whitney U-test. Similarly, the effects of the breed and sex of animals were assessed by comparing the proportions of infected and uninfected animals at each time point using the two-sample test for equality of proportions, with continuity correction. Differences between groups were considered significant at *P* < 0.05.

## Results

### Trypanosome species identified in cattle

To determine the dynamics of trypanosome species in the two cattle cohorts, blood samples from all 40 cattle were analysed for infecting trypanosome using ITS-based PCR, followed by sequencing. The same individual animals were followed over the time of the study; however, the parasite-positive animals at each time point were different as some previously infected cattle tested negative at subsequent time points and vice versa. At the first time point at the Accra site, 12 cattle were found to be infected, and the parasites were *T. theileri, T. vivax* and *T. simiae* (Table 1). At the subsequent sampling time points, between three to eight cattle were found to be infected with one of the same three species identified at the first time point*.* For the other group of cattle at the Adidome study site, which is a higher transmission area, eight, six, nine, twelve, four and eight cattle were infected at time points 1, 2, 3, 4, 5 and 6, respectively (Table 1). The infecting species identified from time points 1 to 5 were *T. theileri* and *T. vivax* while cattle were infected with *T. vivax* at time point 6.

**Table 1:** Trypanosome species identified by nested PCR and sequencing

Time point	Adidome		Accra	
	Number of cattle infected	Trypanosome species detected	Number of cattle infected	Trypanosome species detected
1	8	5 *Trypanosoma vivax* 3 *Trypanosoma theileri*	12 (5)*	6 *Trypanosoma vivax* 9 *Trypanosoma theileri* 2 *Trypanosoma simiae*
2	6 (1)*	6 *Trypanosoma vivax* 1 *Trypanosoma theileri*	8	*Trypanosoma theileri*
3	9	5 *Trypanosoma vivax* 4 *Trypanosoma theileri*	3	*Trypanosoma theileri*
4	12 (1)*	5 *Trypanosoma vivax* 8 *Trypanosoma theileri*	8	*Trypanosoma theileri*
5	4	1 *Trypanosoma vivax* 3 *Trypanosoma theileri*	6	*Trypanosoma theileri*
6	8	*Trypanosoma vivax*	8	1 *Trypanosoma vivax* 7 *Trypanosoma theileri*

*Values in parenthesis represent number of mixed infections

Overall, three species of trypanosomes, namely *Trypanosoma vivax*, *T. theileri* and *T. simiae,* were identified from the study. *Trypanosoma vivax* was the most predominant trypanosome species at the Adidome study site while *T. theileri* was the most predominant species detected at the Accra study site. Both trypanosome species were however detected at the two study sites. *T. simiae* was the least common species as it was detected only at time point 1 at the Accra study site. Mixed infections with two different trypanosome species were detected at time point 1 (four *T. theileri* and *T. vivax* mixed infections and one *T. theileri* and *T. simiae* mixed infection) in Accra and at time points 2 (one *T. theileri* and *T. vivax* mixed infection) and 4 (one *T. theileri* and *T. vivax* mixed infection) in Adidome.

At Adidome, cattle were treated with Diminazene diaceturate 7 weeks prior to time point 1 sample collection. Cattle were also treated between time points 2 and 3, four weeks prior to time point 3 sample collection. However, infections were still detected in some cattle even after the treatment.

### Effect of age, sex and breed on serum biochemical parameters and cytokines

To assess the potential effects of age on the measured serum parameters, the median ages of infected animals at each time point were compared. There were no statistically significant differences between infected and uninfected animals at any of the six-time points, and this was the case at both study sites (*P* > 0.05 in all cases, Mann-Whitney U-test). Thus, the age of animals included in the study will most likely not be a confounder when comparing the levels of serum biochemical parameters and cytokines between infected and uninfected animals.

Likewise, the potential effect of the animal breed was assessed by comparing the respective proportions of animals of each breed per time point for the Adidome and Accra study sites, respectively. Again, there were no significant differences in the proportions of each breed amongst the infected and uninfected animals. Similar comparisons were made for the sex of study animals, and except for the significantly greater proportion of heifers that were infected at time point 1 at the Adidome site (*P* = 0.03, two-sample test for equality of proportions), the proportions of heifers and bulls for all other sampling time points at this study site and all time points at the Accra study site were not significantly different.

### Serum biochemical parameters in cattle during natural trypanosome infection

To establish the levels of serum biochemical parameters during trypanosome infection, serum concentrations of glucose, creatinine, cholesterol, alkaline phosphatase (ALP), alanine aminotransferase (ALT), total bilirubin and total protein were compared between infected and uninfected cattle at the six time points. Due to variation in the dynamics of infection from one time point to the next (Table 1), comparisons were made at each time point.

**Fig. 1:**
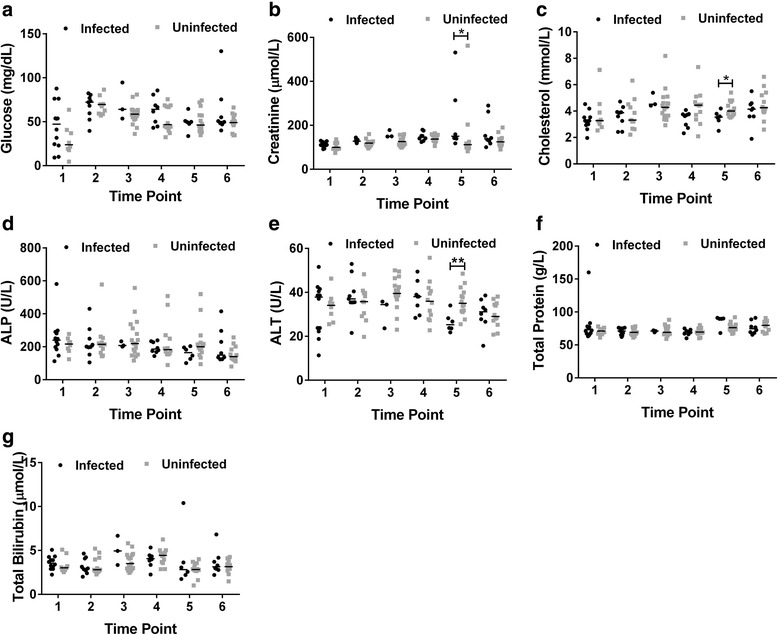
Differences in serum biochemical parameters between trypanosome infected and uninfected cattle in Accra. Levels of **a** glucose **b** creatinine **c** cholesterol **d** ALP **e** ALT **f** total protein **g** total bilirubin was measured. The Mann-Whitney-U-test was used for testing statistical significance between the two groups at each time point. *P < 0.05, **P < 0.01. Time points are at 4 to 5 week intervals

For cattle at Accra, the levels of glucose, ALP, total bilirubin and total protein were not significantly different between infected and uninfected cattle across all six time points (Fig. 1). However creatinine was significantly elevated in infected cattle at time point 5 (infected = 150.2 μmol/l, uninfected = 112 μmol/l; *U* = 11.50, *n*_*1*_ *=* 6, *n*_*2*_ = 14, *P* = 0.01). Cholesterol and ALT were significantly lower in infected cattle at time point 5 (cholesterol: infected = 3.55 mmol/l, uninfected = 4.00 mmol/l, *U* = 16, *n*_*1*_ *=* 6, *n*_*2*_ = 14, *P* = 0.03; ALT: infected = 25.3 U/l, uninfected = 35.0 U/l, *U* = 9.00, *n*_*1*_ *=* 6, *n*_*2*_ = 14, *P* = 0.01, Fig. 1c, e). Levels of these three parameters were not significantly different at all other time points.

**Fig. 2:**
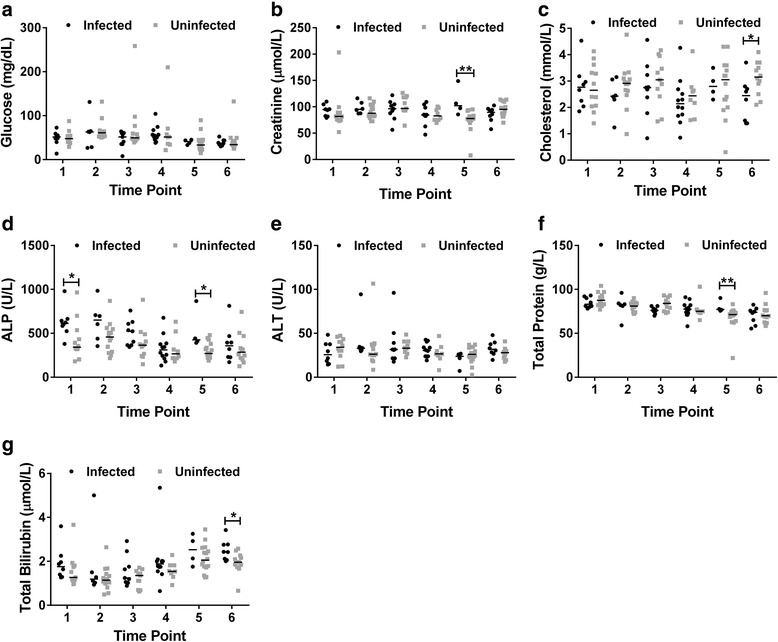
Differences in serum biochemical parameters between trypanosome infected and uninfected cattle in Adidome. Levels of **a** glucose **b** creatinine **c** cholesterol **d** ALP **e** ALT **f** total protein **g** total bilirubin was measured. The Mann-Whitney U-test was used for testing statistical significance between the two groups at each time point, *P < 0.05, **P < 0.01. Time points are at 4 to 5 week intervals

For cattle at Adidome, it was observed that there was no significant difference in the levels of glucose and ALT across all the 6 time points (Fig. 2a, e). ALP was however significantly elevated in trypanosome infected cattle at time point 1 (infected = 613 U/l, uninfected = 342 U/l, *U* = 18.00, *n*_*1*_ *=* 8, *n*_*2*_ = 12, *P* = 0.02) and time point 5 (infected = 462 U/l, uninfected = 271 U/l, *U* = 8.00, *n*_*1*_ *=* 4, *n*_*2*_ = 16, *P* = 0.03) (Fig. 2d). Cholesterol levels were not statistically different between infected and uninfected cattle from time point 1 to time point 5. However at time point 6, infected cattle had significantly lower levels of cholesterol (infected = 2.45 mmol/l, uninfected = 3.15 mmol/l, *U* = 21.00, *n*_*1*_ *=* 8, *n*_*2*_ = 12, *P* = 0.04) (Fig. 2c). Total bilirubin was also not significantly different from time point 1 to time point 5 but at time point 6, infected cattle had significantly higher levels of bilirubin compared to uninfected cattle (*U* = 18.00, *n*_*1*_ *=* 8, *n*_*2*_ = 12, *P* = 0.02, Fig. 2g). Median values of total bilirubin were 2.45 μmol/l and 1.96 μmol/l for infected and uninfected cattle respectively at time point 6. Creatinine was significantly elevated in infected cattle only at time point 5 (infected = 102 μmol/l, uninfected = 77.8 μmol/l, *U* = 3.00, *n*_*1*_ *=* 4, *n*_*2*_ = 16, *P* = 0.01). Total protein was also significantly higher in infected cattle at time point 5 only (infected = 77.1 μmol/l, uninfected = 71.3 μmol/l, *U* = 4.00, *n*_*1*_ *=* 4, *n*_*2*_ = 16, *P* = 0.01).

The levels of serum biochemical parameters were also compared between cattle infected with *T. vivax,* which causes an obvious pathology, and with *T. theileri* which does not. Levels of all measured serum biochemical parameters were similar between *T. vivax* and *T. theileri* infected cattle (*P* > 0.05) (Additional file 2: Figure S1). A comparison of the measured biochemical parameters between mixed and single trypanosome infections also showed no significant difference (Additional file 3: Figure S2).

### Serum levels of pro-inflammatory (IL-12, IFN-γ and TNF-α) and anti-inflammatory (IL-10 and IL-4) cytokines in trypanosome infected and uninfected cattle

**Fig. 3:**
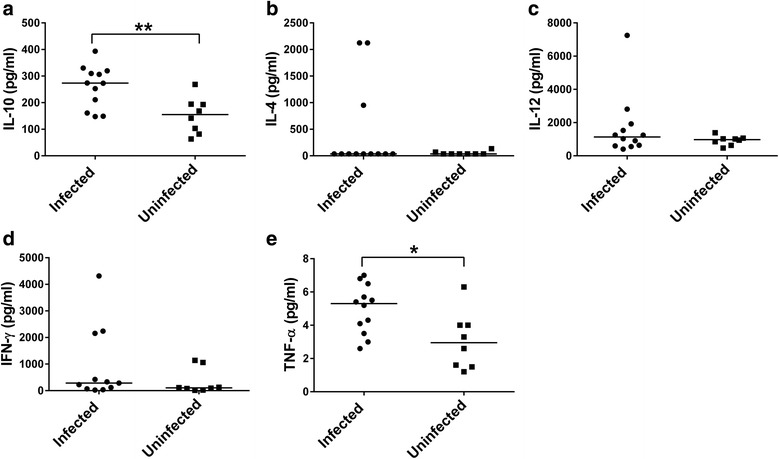
Comparison of serum cytokines between infected and uninfected cattle in Accra. Levels of **a** IL-10 **b** IL-4 **c** IL-12 **d** IFN-γ **e** TNF-α. *Dots* represent individual cytokine values in cattle. Data from time point 1 at Accra were used in the analyses. The *horizontal bar* represents the median. Statistical significance was tested using the Mann-Whitney U-test, *P < 0.05, **P < 0.01

To determine the nature of the immune response that is mounted during natural infection with trypanosomes, serum levels of pro-inflammatory and anti-inflammatory cytokines were compared. Data from time point 1 at Accra and time point 4 at Adidome were used for the cytokine analysis. At the Accra study site, the serum levels of IL-10 and TNF-α were significantly elevated in trypanosome infected cattle compared to the uninfected cattle (Fig. 3). The median concentration of IL-10 was 274 pg/ml (range = 148 pg/ml to 393 pg/ml) in trypanosome infected cattle and 155 pg/ml (range = 63.4 pg/ml to 268 pg/ml) in the uninfected cattle (Mann-Whitney U-test, *U* = 14.00, *n*_*1*_ = 12, *n*_*2*_ = 8, *P* = 0.01). The median concentration of TNF-α was 5.30 pg/ml (range = 2.60 pg/ml to 7.00 pg/ml) in trypanosome infected cattle and 2.95 pg/ml (1.20 pg/ml to 6.30 pg/ml) in uninfected cattle (Mann-Whitney U-test, *U* = 17.50, *n*_*1*_ = 12, *n*_*2*_ = 8, *P* = 0.021). The concentration of IL-10 was higher than the pro-inflammatory cytokines in the infected cattle indicating higher levels of the anti-inflammatory cytokine. The serum levels of IL-4, IL-12 and IFN-γ, were not significantly different between infected and uninfected cattle.

**Fig. 4:**
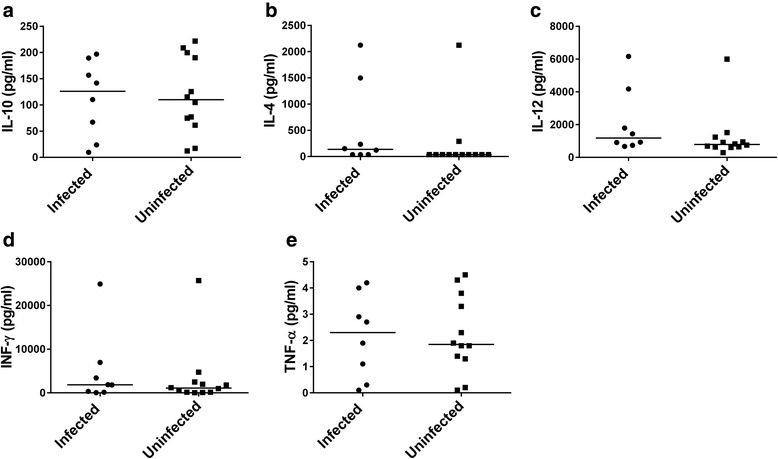
Comparison of serum cytokines between infected and uninfected cattle in Adidome. Levels of **a** IL-10 **b** IL-4 **c** IL-12 **d** IFN-γ **e** TNF-α. *Dots* represent individual cytokine values in cattle. Data from time point 4 at Adidome were used in the analyses. The *horizontal bar* represents the median. Statistical significance was tested using the Mann-Whitney U-test

The levels of IL-10, IL-4, IL-12, IFN-γ and TNF-α measured at the Adidome site showed no significant difference between trypanosome infected and uninfected cattle (Fig. 4).

To determine whether the type of infective trypanosome species have any effect on the levels of cytokine expression, the serum concentrations of pro- and anti-inflammatory cytokines were compared between cattle infected with pathogenic or non-pathogenic trypanosomes, and between cattle with mixed and single trypanosome infections. The serum concentrations of all the measured pro- and anti-inflammatory cytokines were not significantly different in pathogenic (*T. vivax*) trypanosome infection (*n* = 7) compared to non-pathogenic (*T. theileri*) infection (*n* = 5) (Additional file 4: Figure S3). The levels of the cytokines were also similar in mixed and single trypanosome infections (Additional file 5: Figure S4).

### Th1/Th2 cytokine balance in trypanosome-endemic Adidome and non-endemic Accra

To establish the immune profiles that persist during natural infection with trypanosomes, the baseline ratios of pro- to anti-inflammatory (Th1/Th2) cytokines were compared between uninfected cattle in Accra (*n* = 8) and Adidome (*n* = 12) (Additional file 6: Figure S5). Cattle in Adidome had significantly higher IFN-γ/IL-10 cytokine ratios than cattle in Accra (Adidome = 12.4, Accra = 0.91, *U* = 17.00, *P* = 0.02). Also, the IFN-γ/IL-4 cytokine ratios were significantly higher in cattle in Adidome compared to cattle in Accra (Adidome = 19.7, Accra = 1.78, *U* = 21.50, *P* = 0.04). The levels of IL-12/IL-10, IL-12/IL-4, TNF-α/IL-10 and TNF-α/IL-4 were all not significantly different between cattle in the two locations.

**Fig. 5:**
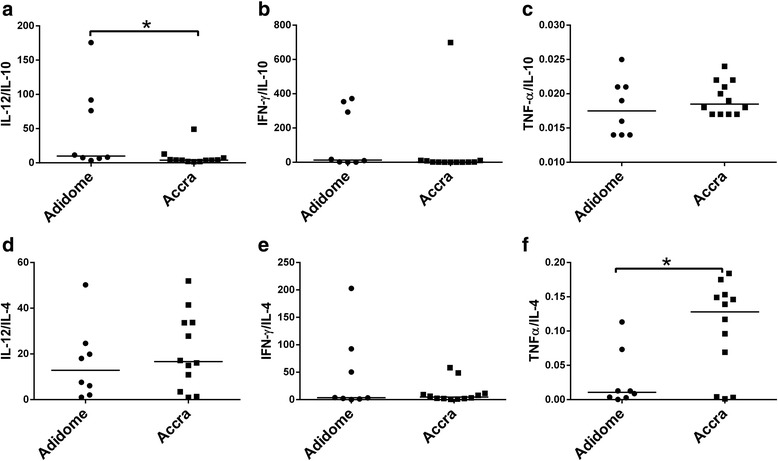
Th1/Th2 cytokine concentration in infected cattle at Adidome and Accra*.* **a** IL12/ IL-10. **b** IL-12/IL-4. **c** INF-γ/IL-10. **d** IFN-γ/IL-4. **e** TNF-α/IL-10. **f** TNF-α/IL-4. Data from time point 1 at Accra and time point 4 at Adidome were used in the analyses. The *horizontal bar* represents the median. Statistical significance was tested using the Mann-Whitney U-test, *P < 0.05

Comparing the pro-inflammatory to anti-inflammatory (Th1/Th2) cytokine ratios between infected cattle in Accra (*n* = 12) and infected cattle in Adidome (*n* = 8), IL-12/IL-4, IFN-γ/IL-10 and IFN-γ/IL-4 were all not significantly different (Fig. 5). IL-12/IL-10 was however significantly higher in infected cattle at Adidome (Adidome = 9.85, Accra = 3.99, *U* = 18.00, *P* = 0.02). TNF-α/IL-4 cytokine ratio was significantly lower in cattle at Adidome compared to Accra (Adidome = 0.01, Accra = 0.13, *U* = 21.00, *P* = 0.04).

### Th1/Th2 cytokine ratios in cattle with the same state of infections over six time points

**Fig. 6:**
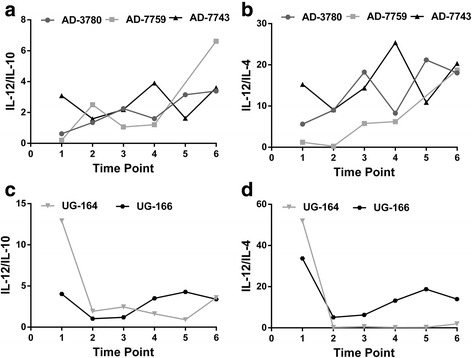
Cytokine ratios in individual cattle at Adidome and Accra over six time points*.* **a** IL-12/ IL-10 levels in two cows (AD-3780 and AD-7759) infected throughout all six time points and a cow (AD-7743) uninfected throughout all the time point at Adidome. **b** IL-12/IL-4 levels in two cows (AD-3780 and AD-7759) infected throughout all six time points and a cow (AD-7743) uninfected throughout all the time point at Adidome. **c** IL-12/ IL-10 levels in two cows (UG-164 & UG-166) infected throughout all six time points in Accra. **d** IL-12/IL-4 levels in two cows (UG-164 & UG-166) infected throughout all six time points in Accra. Time points are at 4 to 5 week intervals

Variation in pro- to anti-inflammatory cytokine response was assessed in individual cattle that had the same state of infection, either infected or uninfected, throughout all the six time points. In cattle at Adidome (AD-3780 and AD-7759) infected throughout the six time points, increasing trends in IL-12/IL-10 and IL-12/IL-4 cytokine ratios were observed (Fig. 6a, b). Although the cytokine ratios fluctuated at some time points, comparing the ratios at time point 1 to time point 6, there was generally an increase in the ratios of IL-12/IL-10 (for AD-3780, time point 1 = 0.620, time point 6 = 3.39; For AD-7759, time point 1 = 0.200, time point 6 = 6.62) and IL-12/IL-4 (for AD-3780, time point 1 = 5.61, time point 6 = 18.0; for AD-7759, time point 1 = 1.22, time point 6 = 18.7). In cow AD-7743, which was uninfected throughout all six time points, however, the IL-12/IL-10 cytokine ratio was not very different comparing the value at time point 1 (3.09) to time point 6 (3.60). IL-12/IL-4 was also not very different comparing the value at time point 1 (15.3) to time point 6 (20.3).

In cattle at Accra (UG-164 and UG-166) infected throughout the six time points, decreasing trends in IL-12/IL-10 and IL-12/IL-4 cytokine ratios were observed (Fig. 6c, d). Although fluctuations in the cytokine ratios were observed at some time points, comparing the ratios at time point 1 to time point 6, a general decrease in the ratios of IL-12/IL-10 (for UG-164, time point 1 = 12.9, time point 6 = 3.59; for UG-166, time point 1 = 4.03, time point 6 = 3.39) and IL-12/IL-4 (for UG-164, time point 1 = 51.9, time point 6 = 1.91; for UG-166, time point 1 = 33.7, time point 6 = 14.0) were observed.

## Discussion

In Accra, trypanosomiasis is less endemic, and *Trypanosoma theileri* was identified as the predominant infecting trypanosome species. *T. theileri* as a non-pathogenic parasite is well tolerated by cattle [22] and therefore may not cause any significant pathology associated with trypanosome infection. This could be the reason for the similar levels of serum biochemical parameters observed in infected and uninfected cattle at most time points at the Accra study site. However, *T. theileri* although largely commensal has been implicated in tissue inflammation [23], hence alterations in some serum biochemical parameters as seen at time point five could be due to the parasite infection. Infection with non-trypanosome parasites such as *Babesia* spp. may further account for the unusual serum titres observed at this time point. Also, trypanosome infection in animals often progresses to chronic states, and during early chronic infections, most serum biochemical parameters drop to normal or near normal values [24, 25]. Chronicity may thus account for the non-significant differences in biochemical parameters observed in cattle at Accra across all six time points. As chronic infections are usually characterised by very low parasitaemia [26], and *T. theileri* infection is known to manifest at low levels throughout the life of the host [22], the high prevalence of *T. theileri* infections at Accra suggests chronic trypanosome infection in cattle at this study site.

At Adidome, a trypanosome endemic area with predominant *T. vivax* infections, high transmission of trypanosomes occurs. The significant differences in biochemical parameters observed at most time points between infected cattle and uninfected controls in this area could thus be due to repeated infection with different *T. vivax* parasites or a rise in parasitaemia because of new antigenic types of parasites unrecognised by the host immune system [27]. Similarities in some biochemical parameters at a few time points may be a result of cattle being chronically infected or having sub-microscopic infections. Also, periodic treatment of cattle with anti-trypanosomal drugs at Adidome could account for this observation. ALP, creatinine, total protein and total bilirubin were significantly elevated in trypanosome-infected cattle whereas cholesterol was significantly reduced. These observed changes are consistent with reports from studies in cattle and other animals [6, 8, 9, 27, 28]. The significantly elevated levels of ALP in infected cattle at time points one and five could be attributed to some factors. ALP is a leakage enzyme usually found in the bone, the intestines and predominantly in the liver [29]. The elevated levels of ALP in the infected cattle, therefore, suggest a probable invasion of the vital body organs and inflammation particularly of the intestines and liver [29, 30]. The high levels of ALP in the infected group may also be due to the destruction of trypanosomes by the host immune system [31]. Several studies have reported significantly elevated ALP levels in both clinical and sub-clinical trypanosome infections [6, 12, 32].

Increased levels of serum creatinine during trypanosome infections have been linked to damage to host tissues or renal malfunction [33]. Elevation in total bilirubin levels in infected cattle as observed in this study is suggestive of hemolytic anaemia due to trypanosomes in blood or jaundice as previously reported in some trypanosome-infected animals [6]. For total protein levels, some studies have demonstrated decreased levels in trypanosome-infected animals; however, these reports have all been linked to acute infections where feed intake is reduced in animals due to illness [11]. Chronically infected animals, however, tend to have increased total protein levels due to increase in gamma globulins produced as part of host immune response [11, 13]. The decreased cholesterol levels observed in infected cattle in this study is consistent with findings in cattle [34], pigs [28] and sheep [35]. The high-energy demand of trypanosomes in infected animals and impaired synthesis or release of cholesterol from the liver, due to pathologic changes induced by trypanosome infection could be responsible for the decreased serum cholesterol levels [28, 36].

The similarity observed in serum biochemical parameters in pathogenically and non-pathogenically infected cattle; as well as in mixed and single species trypanosome infections, implies that regardless of the infecting trypanosome species or the type of trypanosome infection (mixed or single), the levels of serum biochemical parameters do not differ. Similar observations were made in studies in dogs and pigs experimentally infected with African trypanosomes [8, 9].

This study also investigated immune response concerning cytokine profiles in naturally infected cattle at the two study sites. From the study, IL-10 and TNF-α were elevated in trypanosome infections in cattle at Accra. Both IL-10 and TNF-α have been shown to play essential roles in immune response to trypanosome infections. TNF-α is involved in both parasite control and infection mediated pathology [37]. Mice deficient in TNF-α showed increased parasitaemia in *T. brucei* infections [37]. Also, TNF-α has been shown to be involved in reduced T-cell responses in acute infections [17]. IL-10, on the other hand, is an anti-inflammatory cytokine that is known to play a crucial role in resistance to African trypanosomes by downregulating excessive production of pro-inflammatory cytokines [38, 39]. This effect results in reduced pathology associated with immune response. Upregulation of IL-10 in the infected cattle, therefore, suggests a possible down-regulation of Th1 cytokine production mediate immunopathology. Since elevated levels of the Th2 cytokine IL-10 is associated with chronic infections [17, 40], the infected cattle at Accra are most likely experiencing chronic infections supporting the unchanged levels of serum biochemical parameters in infected cattle. The predominance of *T. theileri* and the low transmission intensity of trypanosomes in Accra could be responsible for the chronicity and hence high anti-inflammatory cytokine response. Comparing cytokines between cattle infected with different species and types of infections (single or mixed), it was observed that immune response was not dependent on the infecting species or the type of infection. In a similar study in Uganda, no significant differences were observed in plasma levels of IFN-γ, TNF-α, IL-10 and TGF-β between cattle with mixed and single trypanosome infections; and between cattle infected with trypanosomes of different pathogenicity (Katiti, unpublished observations). At the Adidome study area, there was no difference in individual cytokines compared between infected and uninfected cattle.

In many immune-related disease conditions, the Th1/Th2 cytokine ratio has been shown to be an important indicator of infection and disease outcome. The relevance of the balance between pro- and anti-inflammatory cytokines has been demonstrated in both infectious [41, 42] and non-communicable diseases [43, 44]. On this basis, the pro- to anti-inflammatory cytokine ratios were therefore assessed at basal levels and during infection. Significantly higher pro-inflammatory to anti-inflammatory (Th1/Th2) cytokine ratios were observed in cattle at the Adidome site alone. This indicates a predisposition to higher Th1 immune response. As cattle in this area are treated every two to three months with anti-trypanosomal drugs, clearance of parasites may occur but only for a limited period since the drug is commonly used for curative purposes and offers protection against the disease for only a few days to few weeks when administered [45]. Due to the high transmission intensity in this area, cattle may be re-infected with parasites of different species or parasite strains of the same species [46] expressing different antigenic coats. This could probably be the reason for the high pro-inflammatory cytokine response as cattle are repeatedly exposed to new/different antigens, and hence more likely to carry acute infections. This is supported by observations in cattle that remained infected throughout all six time points. In Adidome where the infecting species differed at some time points, an increasing trend in pro-inflammatory (Th1) immune response was observed from time point one to time point six. However, cattle in Accra infected with the same species at all the time points had an increasing anti-inflammatory immune response from time point one to time point six. Furthermore, modulation of host immune response following berenil treatment [47] at Adidome could also account for the differences in cytokine profiles between cattle at the two study sites. These findings collectively suggest that cattle in an endemic area repeatedly infected with parasites of different species and possibly different antigenic types demonstrate high pro-inflammatory (Th1) immune response whereas cattle in a non-endemic area with chronic infections demonstrate high anti-inflammatory (Th2) immune response.

Some confounding factors such as other haemoparasitic infections and worm infections may have affected the individual measurements of serum biochemical parameters and cytokines in the cattle. However comparing uninfected and infected cattle exposed to the same conditions, controls for these confounders to some extent. Parasite density was however not determined due to failure to detect parasites by microscopy. Another limitation of the study was the routine treatment of cattle at Adidome with anti-trypanosomal drugs.

## Conclusion

This study demonstrates a complicated and much more dynamic biochemical alterations and cytokine production during natural infections. The findings demonstrate that cattle in the non-endemic area, which were predominantly infected with non-pathogenic *T. theileri*, have different biochemical profiles and immune responses compared to cattle in the endemic area with predominantly pathogenic trypanosome (*T. vivax*) infections; suggesting higher biochemical aberrations and pro-inflammatory response in areas with high *T. vivax* infections. Exploring the biomarker potential of biochemical parameters and cytokines will, therefore, require careful consideration of the stage of infection (acute or chronic), endemicity/transmission intensity of an area and treatment practices by livestock farmers.

## Additional files

Additional file 1: Table S1. Characteristics of selected cattle at Accra and Adidome. (XLSX 10 kb)

Additional file 2: Figure S1. Serum biochemical parameters in pathogenic and non-pathogenic trypanosome infections. Levels of (**a**) glucose (**b**) creatinine (**c**) cholesterol (**d**) ALP (**e**) ALT (**f**) total protein (**g**) total bilirubin was measured. The Mann-Whitney U-test was used for testing statistical significance between the two groups. (TIFF 45 kb)

Additional file 3: Figure S2. Serum biochemical parameters in mixed and single trypanosome infections. Levels of (**a**) glucose (**b**) creatinine (**c**) cholesterol (**d**) ALP (**e**) ALT (**f**) total protein (**g**) total bilirubin. The Mann-Whitney U-test was used for testing statistical significance between the two groups. (TIFF 47 kb)

Additional file 4: Figure S3. Comparison of serum cytokines between pathogenic and non-pathogenic trypanosome infections. Levels of (**a**) IL-10 (**b**) IL-4 (**c**) IL-12 (**d**) IFN-γ (**e**) TNF-α. Data from time point 4 at Adidome were used in the analyses. The horizontal bar represents the median. Statistical significance was tested using the Mann-Whitney U-test. (TIFF 48 kb)

Additional file 5: Figure S4. Comparison of serum cytokines between cattle with single and mixed trypanosome infections. Levels of (**a**) IL-10 (**b**) IL-4 (**c**) IL-12 (**d**) IFN-γ (**e**) TNF-α. Data from time point 1 at Accra were used in the analyses. The horizontal bar represents the median. Statistical significance was tested using the Mann-Whitney U-test. (TIFF 50 kb)

Additional file 6: Figure S5. Baseline Th1/Th2 cytokine concentration in cattle at Adidome and Accra. Levels of (**a**) IL12/ IL-10 (**b**) IL-12/IL-4 (**c**) INF-γ/IL-10 (**d**) IFN-γ/IL-4 (**e**) TNF-α/IL-10 (**f**) TNF-α/IL-4. Data from time point 1 at Accra and time point 4 at Adidome were used in the analyses. The horizontal bar represents the median. Statistical significance was tested using the Mann-Whitney U-test, *P < 0.05. (TIFF 57 kb)

## Abbreviations

ALP: Alkaline phosphatase
ALT: Alanine aminotransferase
AST: Aspartate aminotransferase
IFN-γ: Interferon gamma
IL-10: Interleukin 10
IL-12: Interleukin 12
IL-13: Interleukin 13
IL-4: Interleukin 4
IL-6: Interleukin 6
ITS: Internal transcribed spacer
NO: Nitric oxide
TGF-β: Transforming growth factor beta
Th1: Type 1 T helper
Th2: Type 2 T helper
TNF-α: Tumour necrosis factor alpha
